# Blast Testing Issues and TBI: Experimental Models That Lead to Wrong Conclusions

**DOI:** 10.3389/fneur.2015.00072

**Published:** 2015-04-08

**Authors:** Charles E. Needham, David Ritzel, Gregory T. Rule, Suthee Wiri, Leanne Young

**Affiliations:** ^1^Southwest Division, Applied Research Associates, Inc., Albuquerque, NM, USA; ^2^Dyn-FX Consulting Ltd., Amherstburg, ON, Canada; ^3^Security Engineering and Applied Sciences Sector, Applied Research Associates, Inc., San Antonio, TX, USA; ^4^Security Engineering and Applied Sciences Sector, Applied Research Associates, Inc., Dallas, TX, USA

**Keywords:** blast physics, shock tubes, scaling, neurotrauma

## Abstract

Over the past several years, we have noticed an increase in the number of blast injury studies published in peer-reviewed biomedical journals that have utilized improperly conceived experiments. Data from these studies will lead to false conclusions and more confusion than advancement in the understanding of blast injury, particularly blast neurotrauma. Computational methods to properly characterize the blast environment have been available for decades. These methods, combined with a basic understanding of blast wave phenomena, enable researchers to extract useful information from well-documented experiments. This basic understanding must include the differences and interrelationships of static pressure, dynamic pressure, reflected pressure, and total or stagnation pressure in transient shockwave flows, how they relate to loading of objects, and how they are properly measured. However, it is critical that the research community effectively overcomes the confusion that has been compounded by a misunderstanding of the differences between the loading produced by a free field explosive blast and loading produced by a conventional shock tube. The principles of blast scaling have been well established for decades and when properly applied will do much to repair these problems. This paper provides guidance regarding proper experimental methods and offers insights into the implications of improperly designed and executed tests. Through application of computational methods, useful data can be extracted from well-documented historical tests, and future work can be conducted in a way to maximize the effectiveness and use of valuable biological test data.

## Introduction

As explained from the earliest studies of blast physics (1–3), there is a partitioning of energy in a propagating explosive blast wave between static pressure, which inflicts crushing action, and dynamic pressure, which imparts drag and possibly lift forces and is largely responsible for displacement of objects. This energy partition changes with distance: very near the fireball static and dynamics pressures are about equal, but the relative component of dynamic pressure decreases with distance such that as the wave decays to acoustic levels, the induced particle velocity, and hence dynamic pressure becomes negligible. Furthermore, in some cases, it is important to account for the negative phase of the blast wave, during which the blast pressure and flow velocity decay to below ambient levels before gradually returning to ambient. It is important to note that “negative” flow velocity does in fact mean the flow will reverse and material can be swept back toward the blast source.

During an encounter with an object, the blast wave envelops the target and will cause highly spatially and time-variant loading pressure due to the combined effect of the static and dynamic pressure of the incident wave interacting with the target geometry. The “wrap-around” effect of the blast loading is critical to target response in the form of imparted stresses and global displacement.

All of these phenomena can be greatly corrupted in shock-tube experiments, if not very carefully staged. First, conventional shock tubes do not intrinsically generate a “blast-like” shock wave unless the test station is expertly located, and a specimen will be subjected to repeated late-time reflections due to the waves reverberating up and down the length of the tube. Examples of other typical problems include: the measurement of static and dynamic pressure conditions that define the incident shockwave, blockage of the specimen within the tube, failure to allow “wrap-around” loading, inappropriate mounting of specimens, and use of credible scaling rules for models.

## Definitions

To assist with understanding of blast environment, we include here some basic definitions needed for discussing air blast and air blast loading.

*Overpressure* is also known as side-on pressure, static pressure, or gauge pressure. It is defined as the gas pressure, above ambient, which is caused by compression or heating of the gas. The units are force per unit area or energy per unit volume.

*Dynamic pressure* is also known as gust or differential pressure. It is defined as the pressure caused by motion of the gas and is = 1/2 ρ × *U*^2^, where ρ is the gas density and *U* is the gas velocity. Dynamic pressure is a vector which retains the direction of flow as well as the magnitude. The units are the same as overpressure.

*Stagnation pressure* is also known as pitot pressure, total pressure, or total head pressure. It is sometimes confused with reflected pressure. It is defined as the sum of the overpressure and the dynamic pressure. It is the force per unit area that an object in a steady flow experiences.

*Reflected pressure* is the pressure caused by the reflection of a shock wave from a non-responding surface. It is at maximum when the incident shock velocity is perpendicular to the reflecting surface, but is not a monotonic function of the incident angle.

*Overpressure impulse* is the calculated area under the curve of the overpressure versus time function. Classical impulse is a product of the net force applied and the time over which it is applied, and is normally calculated to determine the induced change in momentum of the target. However, in blast physics, the overpressure impulse is defined as the area under the pressure (force per area) versus time or duration function and is calculated by integrating the overpressure as a function of time. In the blast context, impulse does not determine momentum change, but refers to the total energy in a blast wave.

*The symbol* **γ** is the ratio of the specific heat of a gas at constant pressure over the specific heat of the same gas at constant volume. This is an important gas constant in thermodynamic equations and is a critical factor when using different driver gases in shock tube testing.

## Shock Tube Experimental Methods

### Blockage

**Figure 1:**
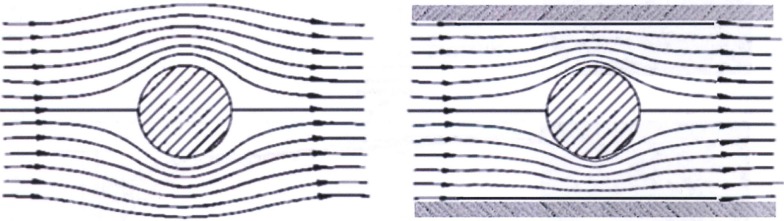
**For studies relevant to quasi-steady flow, the blockage of the specimen should not be >5% of the total cross-section; otherwise, the flow streamline pattern will be overly distorted from the free field as shown at right (Dyn-FX Consulting Ltd.)**.

When experiments are conducted in a shock tube, that is, when the subject is placed within the test section of the shock tube, consideration must be given to the blockage caused by the specimen and its mounting as defined by the ratio of the total “presented area” of the obstruction relative to the cross-section of the tube. The acceptable blockage ratio depends somewhat on nature of the experiment. In classic aerodynamic wind-tunnel testing involving quasi-steady flow, a blockage of 5% or less is recommended. In such testing, the target loading is entirely governed by the free field dynamic pressure causing the forces of lift and drag; it is required to replicate the flow streamline pattern as would develop in “free-flight” conditions. As shown in Figure 1, any confinement will distort this flow pattern and therefore the measured forces of lift and drag as well as the “natural” boundary layer, turbulence, and vortex phenomena that might develop on the object.

Similarly, studies of blast-induced drag give the equation (4):

$$\mathrm{Qb}=Q0\left[\exp\left(2.64\times R^{1.038}\right)\right]$$

where Qb is the dynamic pressure in the partially blocked tube, Q0 is the dynamic pressure with no blockage, and *R* is the blockage ratio (the cross sectional area of the target divided by the cross sectional area of the shock tube test section). Evaluating this equation at 10% blockage, the dynamic pressure (and the drag load) is increased by 27% over the empty tube value. At 20% blockage, the dynamic pressure is increased by 64% and at 30% it is increased by 113%. This has been known and published for over 30 years, yet experiments continue to be conducted in shock tubes that are much too small for the test subjects.

The criteria above relate to blast encounters where the blast response is dominated by drag/lift forces of quasi-steady flow, i.e., the blast wavelength (λ, roughly the duration multiplied by the speed of sound) is long compared to the characteristic length of the target (*L*, which might be diameter of a sphere). For example, λ ≫ *L* in the case of a nuclear blast wave incident on vehicles or personnel.

However, when the blast damage or injury is dominated by the initial blast reflection and diffraction of the shock front around the object, somewhat different criteria may apply. Wortman and Lottero (5) showed that for λ ~ *L*, a less-stringent criteria of 20% blockage was acceptable due to the rapidly decaying nature of the dynamic pressure. For experiments with inanimate objects, it is possible to accept even higher blockage by “cutting off” the relevant data-capture prior to the arrival of the shock reflection from the wall of the tube. However, animal specimens will of course accumulate injury from the entire history of exposure in a shock tube, including late-time reflections from the end-condition of the tube and even the ingress of driver-gas into the test section in the seconds following the shock exposure. Therefore, it is critical to assess the damage mode of relevance in the staging of an experiment, which in turn relates to a hypothesis for the injury mode and scaling criteria discussed later. It may be that the response of an animal model to a blast wave of 2 ms duration is dominated by “blast throw” forces whereas the response of a human is dominated by shock reflection and diffraction.

The Rankine–Hugoniot relations are a statement of the conservation equations at the shock front for a blast wave. They have very general applicability and can be used in a simplified form for blast pressures below 2,000 kPa. These relations are very useful because they can provide all of the shock front parameters given any one of the shock values and the ambient atmospheric conditions through which the shock is moving. For example, a shock having overpressure Δ*P* in an ambient atmosphere of pressure *P*_0_ has a dynamic pressure (*q*) of:

$$\mathrm{Dynamic Pressure}=q=\frac{5}{2}\times \frac{\Delta P^{2}}{7P_{0}+\Delta P}$$

The stagnation pressure is Δ*P* + *q* and the reflected pressure is given by:

$$P_{\mathrm{ref}}=2\Delta P+\left(\gamma +1\right)q$$

where γ is the specific heat ratio for air (γ = 1.4). The expression shows the reflection factor is a minimum of two times the overpressure (Δ*P*) and is even greater depending on the values of γ and *q*. Thus, enhancements in dynamic pressure (caused, for example, by exit jet gassing from a shock tube) lead to reflected pressure loads that may be significantly greater than would be measured in free field loads, even if the overpressure waveforms are similar.

### Addressing the dynamic pressure problem

Some experimenters, who are familiar with the multiple problems caused by shock tube blockage, have added conical expansion tubes to their shock tubes. By placing test subjects in the conical section of the tube, they can alleviate some of the blockage problem, because the test section now has a cross section that is larger and the blockage area is reduced. This change from a fixed cross section to an expanding cross section, however, introduces perturbations to the incident blast wave that must be accounted for when evaluating the experimental data.

Another solution that has been adopted by a number of experimenters is to place the test object outside the tube. While this may appear to be a solution to the blockage problem, the blast phenomena near the exit of the tube is drastically modified by the sudden expansion of the blast wave upon exiting the tube. The sudden expansion causes many changes to the blast environment, including asymmetrical overpressure distribution, formation of a strong vortex flow, enhanced dynamic pressure, and an overall non-uniformity of the loads across the entire cross section of the target.

**Figure 2:**
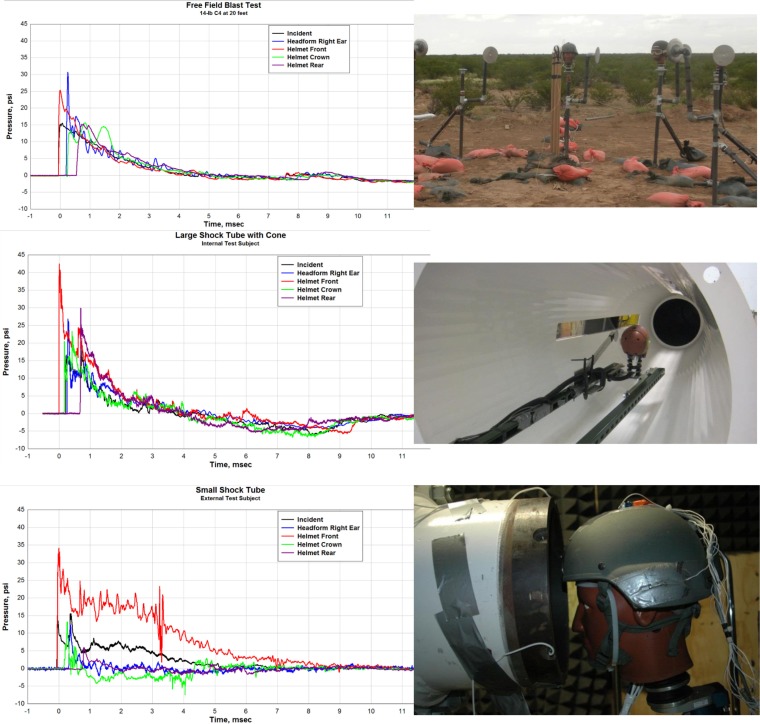
**Free field, conical, and exit jet load waveform comparisons (6)**.

The three waveforms in Figure 2 compare the incident and load pressures on similar targets. In all cases, the incident overpressure is just over 100 kPa and a target duration of about 4 ms. The conical tube does a reasonable job of duplicating the free field environment, but has introduced some secondary shocks. The experiment with the head in the exit jet shows significant deviations from the free field case. The load on the front of the helmet remains high for a time of more than twice the incident duration of the free field case resulting in an artificially enhanced impulse exposure. This is caused by a combination of area blockage and the greatly enhanced dynamic pressure in the “exit jet.” The pressures on the crown (top) and rear of the helmet are reduced, with a shortened positive duration and a long negative phase (6). Such a pressure distribution leads to great overestimations of the total forces and impulses delivered to the target. A surprising number of papers presented in biomedical journals employ end-jet testing exacerbated by high-blockage.

**Figure 3:**
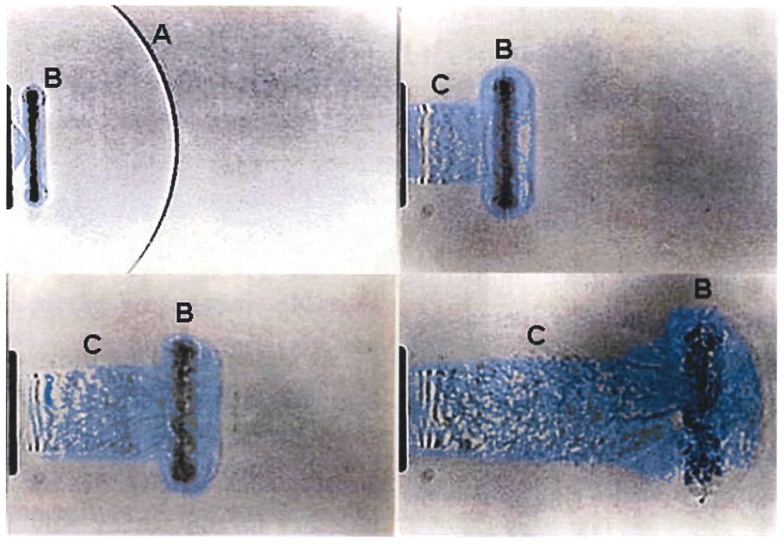
**Time sequence showing development of a shock-tube end-jet (efflux gas artificially colored)**. (A) The muzzle-blast shock front rapidly diffracts, weakens, and separates from the plume; (B) ring vortex develops and separates from the lip of the tube end and is swept along with the venting column of shock-tube gases. (C) The venting jet of high-speed shock-tube gases has extreme dynamic pressure and long duration having an entirely different time waveform than the static pressure condition (7).

In fact, the flow-field that develops from the open end of a shock tube is complex and composed of two distinct regimes: (1) the decaying but quasi-steady efflux of gases formerly within the tube, which forms a collimated jet of roughly the same diameter as the tube, and (2) a propagating diffracting shock front known as the “muzzle blast.” A “smoke-ring” vortex will also develop from the rim of tube as shown in Figure 3. Although the diffracting shock wave might appear spherical, it is in fact very non-uniform with high gradients as a function of angle and distance from the exit. The muzzle blast will have a very much shorter duration than the shockwave within the tube. Objects in-line with the exit jet will be briefly exposed to the diffracting shock, although loading is dominated by the decaying quasi-steady jet which is, in fact, entirely unrelated to conditions of a propagating blast wave.

**Figure 4:**
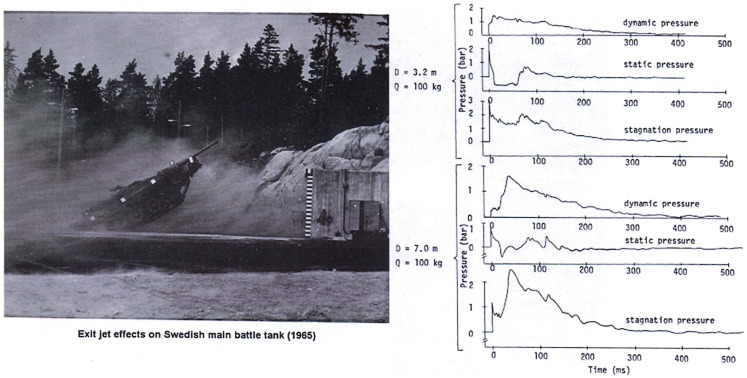
**Displacement of a main battle tank in the anomalous conditions of end-jet flow with vastly exaggerated dynamic pressure as well as periods of vacuum static pressure conditions (8)**.

Experiments conducted at large blast simulators have shown the highly anomalous effects of “end-jet” testing. As shown in Figure 4 from a Swedish study (8), the dynamic pressure impulse in the end-jet can be 100-fold that of a true blast wave having the same static pressure condition. Also, very importantly, the traces show that the static pressure in the jet can fall well below ambient levels for sustained periods, causing anomalous vacuum effects on the target combined with the greatly exaggerated flow velocity.

### Exploiting the exit jets from a shock tube

Exit jets from shock tubes cannot be used to generate blast waves simulating those from free field detonations. They can be very useful if the experimenter is attempting to simulate the enhanced dynamic pressure of a nuclear precursed environment. We have successfully demonstrated the use of exit jet loading to simulate the reduced overpressure, enhanced dynamic pressure, and greatly enhanced dynamic pressure impulse of nuclear blast precursors. There are several notable differences between the ideal or free field blast wave and the measured exit jet wave. The peak dynamic pressure is the result of the acceleration of cold air caused by the sudden expansion of the shock wave at the tunnel exit and is not at the shock front. There are multiple peaks, with the highest being about three times greater than the free field wave, and the dynamic impulse is about a factor of six greater than for the free field.

**Figure 5:**
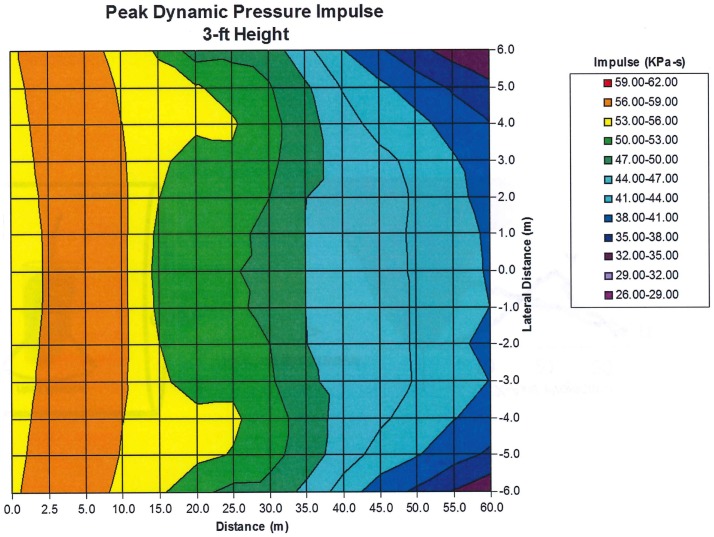
**Dynamic pressure impulse as a function of distance from the tube exit and lateral distance from the center line**.

Because there is a sudden expansion of the shock at the end of the shock tube, rarefaction waves move into the flow from the edge of the tube. This introduces significant non-uniformities to the flow and reduces the useful area in which experiments can be made. To show the variation of the dynamic pressure impulse as a function of position in the exit jet, we include Figure 5. The peak dynamic impulse as a function of distance from the tube exit and the lateral distance across the tube is shown. The peak overpressure measured on the center line at 1.5 tube diameters from the exit is about 70 kPa. The plot covers only the central half of the 20 m diameter of the tube; variations beyond half the radius of the tube (from the center line) are too great to be used in any experiment. Large gradients in the flow are caused by expansion waves initiated at the edge of the end of the tube. These results are characteristic of any shock tube. Therefore, exit jets from shock tubes cannot be used to generate blast waves simulating free field detonations.

### Specimen mounting

**Figure 6:**
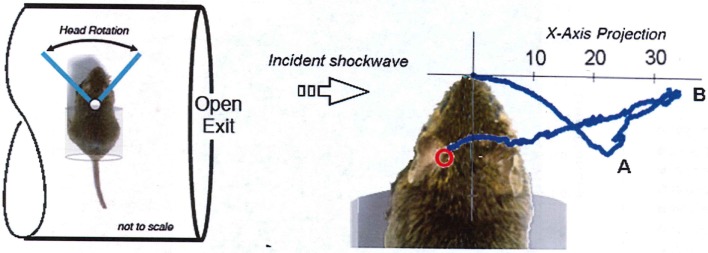
**Extracts from Goldstein showing the mounting canister for a specimen located near the shock-tube exit**. Tracking of the nose of the specimen shows the head made a violent focal impact with the rim of the canister at Point “A,” then rebounded to its maximum extension at Point B before retracting back to the canister (9).

The mounting of a specimen in shock-tube testing must be done with the same care applied for supersonic wind-tunnel research. Apart from the blockage aspect described earlier, anomalous loading of the specimen will be caused by local shock reflections and flow patterns developed around the support structure. There is also high potential for inflicting injury artifacts entirely due to the restraint system. For example, Goldstein (9) describes a cylindrical canister for mounting a rat specimen perpendicular to the shock tube flow as shown in Figure 6. Being located near the exit, the test location will be subjected to exaggerated dynamic pressure exacerbated by high-speed flow around the canister; anomalous pressure loading would be imparted to the body and head due to the canister. Perhaps most importantly, in that case, tracking of the head motion shows the head made a violent focal impact with the rim of the canister.

## Scaling Issues

The examples we have used so far range from small scale shock tube and free field tests to nuclear scale phenomena. Be assured that air blast phenomena scale very well using cube root of the energy for free field parameters. It is important to realize that the duration of the positive phase of the blast loading must be compatible with the size and type of target being tested.

For blast effects from improvised explosive devices and typical munitions, the positive durations at the pressure levels for human injury range from 1 to 10 ms. A low strength blast wave travels at a little more than 1 foot per millisecond. One millisecond is roughly the time that it takes for the shock wave to engulf an erect human. Thus, the range of applicable durations is from the time that it takes to engulf the target to 10 times that duration.

If we take an example of a mouse as the target, we can approximate the dimension of a mouse head to be 10% of the dimension of a human head. The duration of blast waves used with mice should, therefore, be between 0.1 and 1 ms, based on size. However, previously established pulmonary blast scaling rules are based on relative mass. Bowen (10) established the cube-root mass scaling law, where a 30 g mouse loaded for a duration of 1 ms would scale to a 70-kg human equivalent duration of 13 ms. Although this approach to scaling has been used extensively, even for pulmonary injury this method leaves out a number of factors that may have a significant influence on the response of difference species to the same blast load, such as rib stiffness, lung spring constant, and lung density. It remains unclear from Bowen et al.’s tests exactly how body mass directly relates to the injury mechanics of blast lung. When the injury in question is TBI, a new set of factors become potentially relevant, including skull geometry, stiffness, and porosity, among others. Given the lack of understanding necessary to reveal accurate scaling rules, at a minimum, physics principles should apply. The total positive duration for a mouse should not exceed 1 ms for both the overpressure and the dynamic pressure. If a mouse subject is loaded for a duration of 10 ms, the human-equivalent duration is over 100 ms (ten times the maximum reasonable IED blast duration), and the effective yield of the explosion has 1,000 times too much energy.

The caution here is that the blast wave must fit the target or the response will not be meaningful. This also points out that the effective blast yield is proportional to the cube of the duration and is, therefore, a very strong function of the duration. A variation of a factor of two in duration translates to a factor of eight in effective energy in the source of the blast wave. For this reason, too, experiments must be carefully controlled. Special care must be taken to measure the duration of the dynamic pressure as well as the overpressure. In experiments using the exit jet, the dynamic pressure positive phase may be two to three times that of the overpressure and the magnitude of the dynamic pressure can be more than three times that of a free field blast wave. The dynamic load on a test subject in the exit jet as measured by the total impulse of dynamic pressure can well-exceed 10-fold that of a free field blast at the same peak overpressure level.

## Load Interpretation

It has been quite common in biomedical research to undertake a purely empirical approach to injury analyses; i.e., assess the injury outcome of a specified insult condition without detailed understanding of the physics and biomechanics of the injury processes occurring at the cellular scale during the event. This approach was in fact that taken by the Lovelace group in its pioneering studies of blast injury (10), where injury outcomes were mapped against the intensity of the incident blast (peak pressure and duration or impulse). In neurology, this empirical approach was also the basis for the head injury criteria (HIC), and the development of laboratory devices such those for controlled cortical impact and fluid percussion testing. In all such cases, some simplistic global parameters, such as the incident static pressure, global acceleration, or fall of a drop-weight, are used to define a prescribed insult without any detailed understanding of what is happening on the cellular scale.

However, a modern multi-disciplinary approach to injury studies allows the means to understand the detailed biomechanics of an injury event using powerful tools such as computational simulations of the physics. Therefore, rather than treat the injury event as a “black box”, it is possible to resolve the detailed spatial and temporal loading and to determine exactly how that loading imparts stresses on biological tissues on the scale of milliseconds. Biological specimens and especially the human brain are extremely complex structures from both the perspective of geometry and material properties. For such studies, it will usually be required to combine discrete experimental measurements with computational modeling.

**Figure 7:**
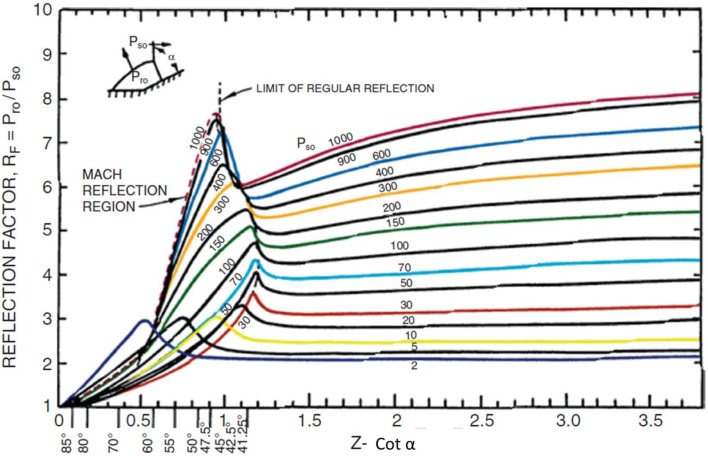
**Reflection factors (RF) as a function of the cotangent (*Z*) of the incident angle (*a*)**. The reflection factor is the ration of peak reflected overpressure (*P*_r0_) to peak incident overpressure (*P*_s0_) (11).

One or two gauges on the test object are insufficient in the absence of computational fluid dynamics (CFD) simulations to fill in the gaps required to define loading over a complex shape such as human head fitted with a helmet. As pointed out earlier, the reflection factor for shock waves is not a monotonic function of the angle of incidence. Figure 7 shows the reflection factor for several incident pressure levels as a function of the cotangent of the incident angle. As the cotangent approaches zero (for a surface parallel to the blast wave), there is no reflection and the load is the incident overpressure. At large values of the cotangent, the reflection factor approaches that of a normal reflection. The enhancement in reflection factor near 45° is the result of the transition from regular reflection to Mach reflection (11).

**Figure 8:**
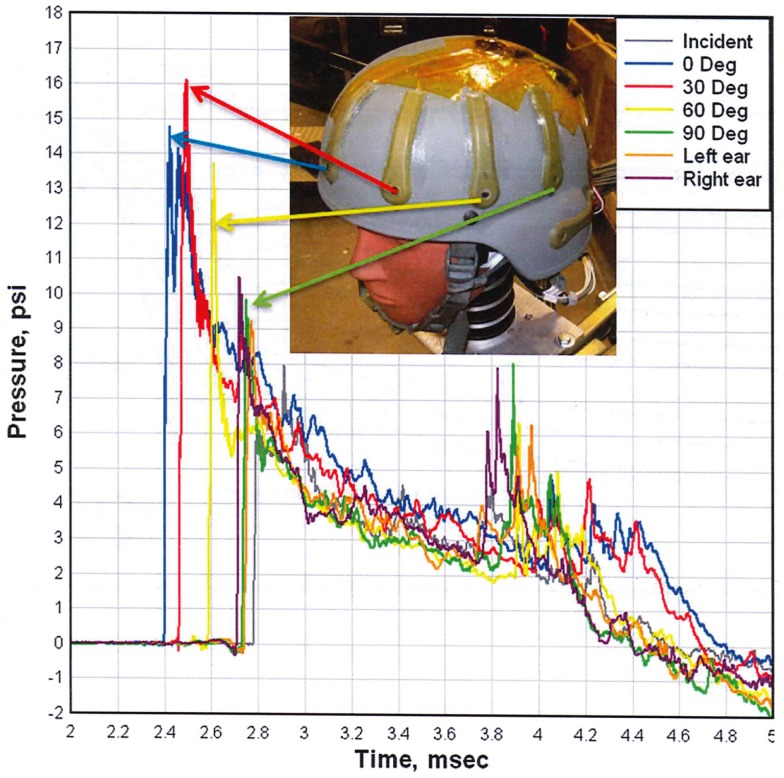
**Comparison of overpressure waveforms measured on a helmeted head (12)**.

To demonstrate the effect of this angular dependency, we include Figure 8, which compares the overpressure waveforms at several positions around a helmeted head in a conical expansion shock tube (12). The peak measured overpressure is greater at the 30° angle than it is at the head on position. The peak overpressure load then decreases rapidly as the shock engulfs the head. The positive duration of the load is nearly 2.5 ms. To calculate the acceleration of the head, the assumption is that the load is vertically uniform, and that each gauge record represents some fraction of the exposed surface area. The impulsive load is calculated by integrating the overpressure waveforms over the positive duration. However, without a clear understanding of the mechanics of injury, the validity of these assumptions cannot be ascertained.

**Figure 9:**
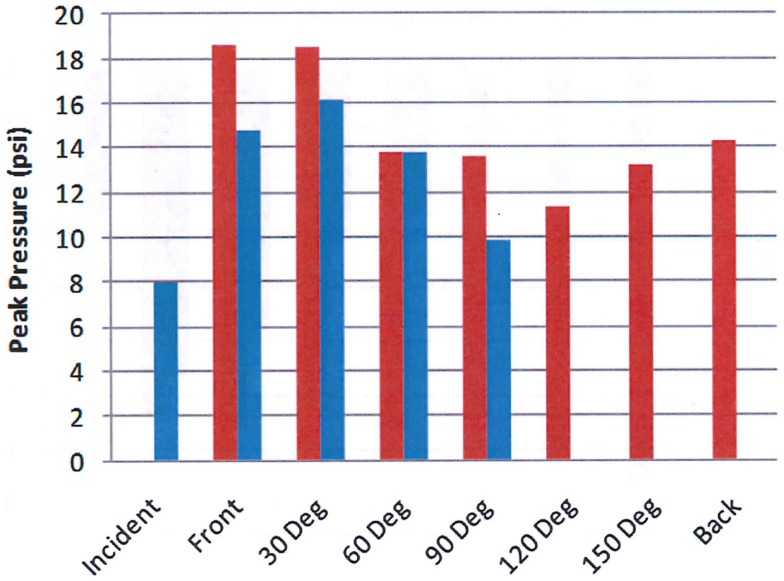
**Calculated (red) and measured (blue) peak overpressure as a function of angle**. The blue bars are measured values from the helmet test shown in Figure 8 and the red bars are calculated overpressure levels using Second-Order Hydrodynamics Automatic Mesh Refinement Code (SHAMRC) (12).

Figure 9 shows the peak overpressure as calculated using the physics-based CFD code, Second-Order Hydrodynamic Automatic Mesh Refinement Code (SHAMRC), and measured as a function of angle around the head using the test set up shown in Figure 8. There are some differences of note. For example, the calculation was done for a free air environment, whereas the experiment was completed within the confines of a shock tube and the simulation was for an explosively generated blast, while the shock tube used compressed air to generate the blast wave. Additionally, the calculation monitored the pressure load at more than 200 locations on the head, but the test data is limited to the four sensors mounted on the helmet at the front, 30°, 60°, and 90° positions. Overpressure comparisons are shown only for those positions closest to the experimental gauges. Note that the calculated peak overpressure on the back of the head is more than three quarters of the peak at the front of the head. This is caused by the convergence of multiple shocks after they pass over and around the head.

**Figure 10:**
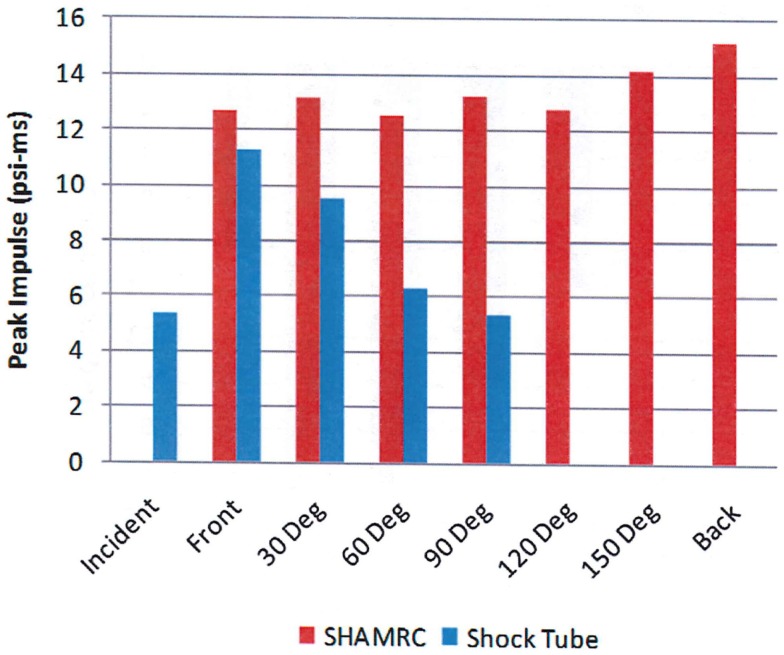
**Calculated (red) and measured (blue) overpressure impulse as a function of angle (12)**.

However, the peak overpressure indicates only a part of the blast load. Figure 10 compares the measured and calculated overpressure impulse at the same positions around the head. It is the impulse that determines the momentum transfer to the test subject. The comparison illustrates the potential difference in overpressure impulse loading between free field blast and a shock tube. Note that the calculated peak overpressure is in good agreement with the experimental data in Figure 9, but the impulses differ significantly due to the differences in the source loading. Also note that the impulse does not correlate with the peak overpressure in either the test data or the simulation. These differences are a result of the blockage in the shock tube, which will have a small effect on the peak overpressure, but can cause significant decreases in the dynamic loads to the sides and back of test objects as previously discussed, resulting in skewed dynamic pressure impulse exposures.

## Implications

The implications of improperly conducted blast test experiments are considerable, particularly in relation to neurotrauma studies where the exact mechanism of blast-induced neurotrauma remains unknown. This applies not only to mechanism studies, but threshold studies as well, where live animals are exposed to shock tube generated blast waves to generate neurotrauma. Some considerations are described below.

- Placement of test subjects inside a tube that is too small: When test subjects block more than 10% of the cross-sectional area of the test section of the shock tube, the resulting excess impulse can increase the effective yield by several times. Furthermore, since a vast majority of the excess energy is a result of the dynamic pressure, there is a potential for head acceleration sufficient to induce brain injuries that may not otherwise have occurred in the absence of elevated dynamic pressures (9). Additionally, the excessive impulse unique to this environment makes existing mass-based or computational-based scaling models (10, 13) inappropriate because the energy from the blast does not follow the same function of peak overpressure and duration.
- Placement of test subjects outside the tube without accurately accounting for the environment: when subjects are placed outside the opening of the shock tube, it is necessary to avoid testing in the jet efflux in-line with the tube axis. The diffracted shock front off-axis from the tube will be of extremely short duration and have high gradients as a function of angle and radial distance from the exit, but is indeed a form of blast wave (14).
- Failure to apply blast scaling laws: this is often seen with rodent test series. When shock tubes are used to load small subjects (e.g., under 1 kg), positive phase durations are frequently reported in the 2–4 ms range. However, depending on the scaling method used, this can translate to human equivalent durations of over ten times that, which can lead to as much as a 1000-fold increase in the effective yield of the shock tube generated blast.
- Insufficient or improperly staged instrumentation: measuring incident static pressures without an understanding of the corresponding dynamic or total pressure and consequent reflected pressures on the test subject can lead to mischaracterization of the effective load that leads to any measured injury outcomes.

## Recommendations for Future Work

We have attempted to point out the major pitfalls of blast testing using shock tubes as publicized in recent biomedical literature particularly regarding the problem of blast-induced neurotrauma. Shock tubes can closely approximate conditions of free field explosive blast if the apparatus and experiments are carefully designed by those knowledgeable in the area of blast physics and experimentation; they offer an efficient means to provide repeatable and consistent results in a controlled, laboratory environment. However, there are number of important considerations in the design and conduct of such experiments to simulate conditions of free field explosive blast. Firstly, the blast loading of models should be scaled in accordance with the ratio of the blast wavelength to the characteristic length of the actual subject (which might be the head diameter for example). When dealing with typical yields from expedient improvised explosive devices, the simulation must have a positive duration that is between 1 and 10 times the dimension of the target. For most pressures of interest for injury studies, the blast wave travels at about 1 foot per millisecond; therefore, a 1 ms pulse is the minimum for a human sized target and a maximum for a mouse sized target. Furthermore, scaling should also be applied with respect to the particular response mode of the specimen hypothesized for the injury mechanism; that mechanism might be blast-induced acceleration or stress imparted by skull flexure, for example.

When designing shock-tube tests, it is very important that blockage effects be considered, although “acceptable” blockage depends on the nature of the experiment and somewhat on the duration of the wave relative to the characteristic length of the target. When the injury study relates to blast-induced motion from drag and the relative shock wavelength is long, the total blockage of the specimen with its mounting should be <10% of the cross sectional area of the test section. When the study concerns the initial reflection/diffraction phase or the relative wavelength is short, blockage as high as 25% may be tolerable, with test time limited by the arrival of the reflected shock from the wall, provided the test specimen is inanimate. In all cases, it is recommended to conduct a CFD study to assess the nature and degree of blockage effect.

Testing in the end-jet flow or within a shock tube and close to the open end has been done for ease of mounting or viewing specimens or, in some cases, simply because the specimen will not fit within the tube. However, end-jet testing will result in anomalous loading unlike that of free field explosive blast, particularly due to the greatly exaggerated dynamic pressure, which can easily exceed 5-fold its proper level, yet is rarely measured. The exaggerated dynamic pressure will show as falsely enhanced “blast throw” effects and will be associated with comparable reduction in static overpressure which causes “crushing” action; indeed periods of high vacuum may exist in the exit jet as well as “standing” recompression shocks. Near the exit plane itself, there are extreme gradients and non-uniform flow conditions; measurements taken just inside the tube near the end are, in fact, entirely different from those outside. Therefore, data on dynamic pressure should be recorded and reported along with the complete waveform plots (rather than just peak overpressure); so, other researchers can understand the nature of the flow field and subject exposure in the experiment.

The study of blast injury in humans is fraught with challenges, since any analysis must be based on a model of some sort, which will likely never have means to be validated. Therefore, the integrity of the injury model and the credibility of its extension to the human case are paramount. Of all injury studies, the investigation of blast-induced mild TBI represents the most extreme of challenges since almost every aspect of the injury remains unknown, from the biomechanics explaining “‘how, where, and what” critical mechanical stress is being imparted to the nature of disruption at the cellular scale. It is not clear, for example, that even cellular disruption or cell-death is necessary for some brain dysfunction to be inflicted. Already there are serious questions raised from the biomedical perspective on the validity of non-primate animal models for any mechanically inflicted brain injury relevant to humans. Any such problems are hugely compounded by invalid test methodology. Yet, in this case, the knowledge for proper laboratory simulation of blast insult and supportive computational modeling have been available for decades. The biophysical implications of invalid blast-simulation in this research are difficult to assess, and it may be that the use of animal models may exaggerate or in fact hide injury outcomes that would be inflicted in humans. For this reason, experimenters are encouraged to design and conduct laboratory tests with input from those experienced in the area of blast physics and experimentation including the use of CFD methods to validate the test configuration. Finally, although often difficult, it is always desirable to validate any simulation with the staging of at least a small set of actual free field explosive blast tests.

## Conflict of Interest Statement

The authors declare that the research was conducted in the absence of any commercial or financial relationships that could be construed as a potential conflict of interest.
